# Hookworms in the Stomach: Concurrent Gastric Ancylostomiasis and Gastric Adenocarcinoma Video Case Report

**DOI:** 10.14309/crj.0000000000002313

**Published:** 2026-09-28

**Authors:** Anil Mathew Philip, Victoria Alagiozian-Angelova, Anas Almoghrabi

**Affiliations:** 1 Department of Internal Medicine, John H. Stroger Hospital of Cook County, Chicago, IL; 2 Department of Pathology and Laboratory Medicine, John H. Stroger Hospital of Cook County, Chicago, IL; 3 Department of Gastroenterology and Hepatology, John H. Stroger Hospital of Cook County, Chicago, IL

## CASE REPORT

A 79-year-old woman with heart failure and hypertension, who traveled between Honduras and the United States, presented with 3 months of epigastric pain, vomiting, diarrhea, and 60-pound weight loss. Evaluation revealed iron-deficiency anemia, eosinophilia (1,200 cells/µL), and a gastric ulcer concerning for malignancy, with retroperitoneal lymphadenopathy and hepatic lesions.

Esophagogastroduodenoscopy revealed a large, ulcerated lesion with a fibrinous base and friable margins along the lesser curvature and incisura (Video 1, Figure 1), confirmed as moderately differentiated gastric adenocarcinoma with a desmoplastic stroma (Figure 2). Translucent, S-shaped worms were also seen attached to the gastric mucosa (Video 1, Figure 3) and duodenum, confirmed as Ancylostoma species on histology (Figure 4).

**Figure 1.**
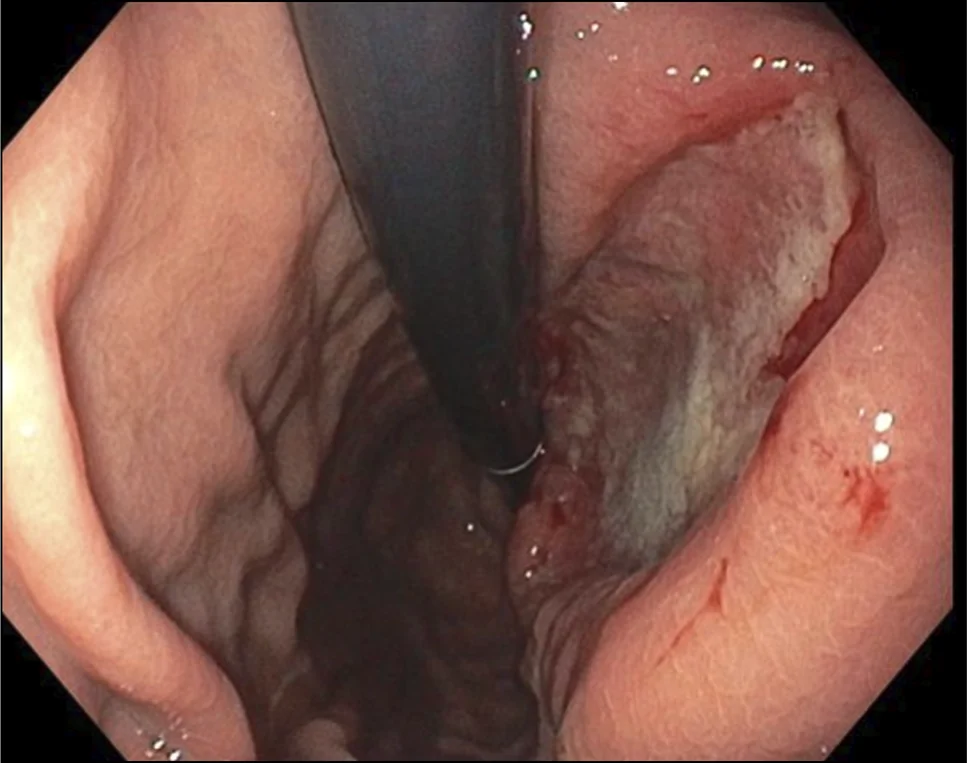
Esophagogastroduodenoscopy demonstrating a large, ulcerated lesion with a fibrinous base and raised, friable margins along the lesser curvature and incisura, subsequently confirmed as gastric adenocarcinoma.

**Figure 2.**
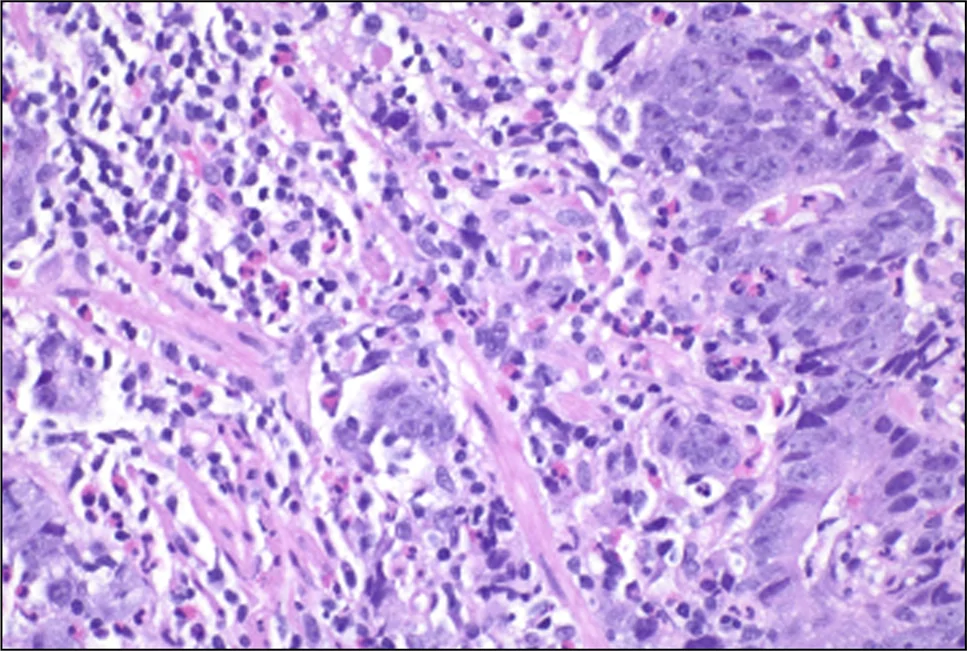
Hematoxylin and eosin stain demonstrating moderately differentiated gastric adenocarcinoma with malignant gland formation infiltrating a desmoplastic stroma (original magnification ×400).

**Figure 3.**
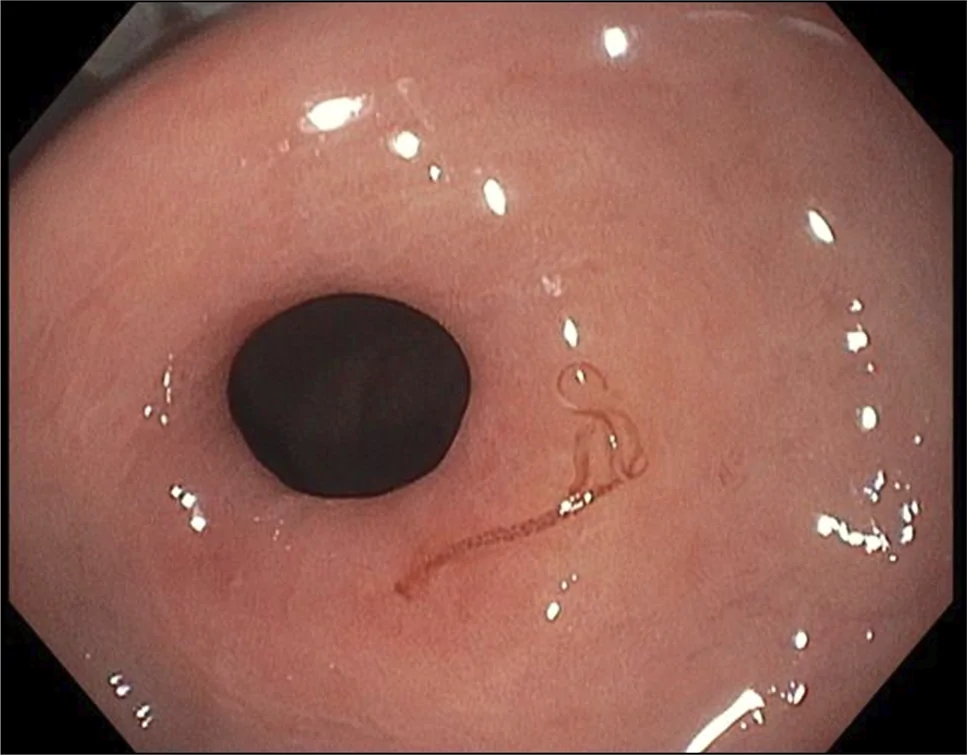
Endoscopic visualization of a translucent Ancylostoma species attached to the gastric mucosa in an S-shaped configuration. Similar organisms were identified in the duodenum.

**Figure 4.**
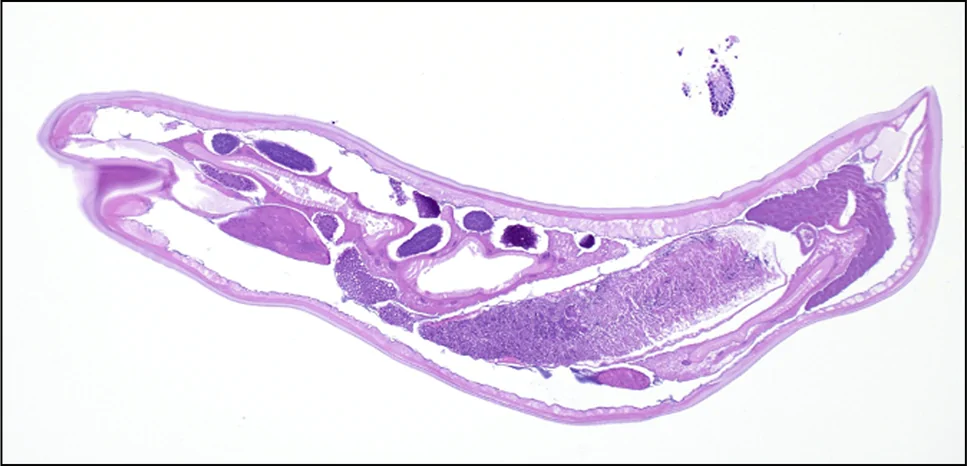
Low-power (×100 magnification) histopathologic image of Ancylostoma species demonstrating an intact nematode cross-section with preserved cuticle and internal structures (hematoxylin and eosin stain).

The patient was treated with albendazole and neoadjuvant chemotherapy, followed by total gastrectomy. Her postoperative course was complicated by multidrug-resistant urosepsis and multiorgan failure; she died 3 weeks later.

Gastric hookworm infestation is rarely visualized endoscopically, as Ancylostoma typically localizes to the small intestine, though upper gastrointestinal involvement is reported.^1,2^ Parasite-malignancy coexistence is similarly uncommon, as with a reported Anisakis larva on early gastric cancer.^3^ This case underscores the importance of considering parasitic coinfection in patients from endemic regions with weight loss, anemia, and eosinophilia, even after a malignant diagnosis has been established.

## DISCLOSURES

Author contributions: AM Philip: manuscript drafting, video and image acquisition, clinical care; V. Alagiozian-Angelova: histopathology interpretation and figures; A. Almoghrabi: endoscopy, clinical care, manuscript review. AM Philip is the article guarantor.

Financial disclosure: None to report.

Informed consent was obtained for this case report.
